# The Role of Noncoding RNA in the Transmission and Pathogenicity of Flaviviruses

**DOI:** 10.3390/v16020242

**Published:** 2024-02-02

**Authors:** Xianwen Zhang, Yuhan Li, Yingyi Cao, Ying Wu, Gong Cheng

**Affiliations:** 1 Shenzhen Bay Laboratory, Institute of Infectious Diseases, Shenzhen 518000, China; 2 New Cornerstone Science Laboratory, Tsinghua University-Peking University Joint Center for Life Sciences, School of Basic Medical Sciences, Tsinghua University, Beijing 100084, China; lyhpluie@163.com (Y.L.); yy-cao22@mails.tsinghua.edu.cn (Y.C.); 3 State Key Laboratory of Virology and Hubei Province Key Laboratory of Allergy and Immunology, Institute of Medical Virology, TaiKang Medical School, Wuhan University, Wuhan 430072, China; yingwu@whu.edu.cn; 4 Institute of Pathogenic Organisms, Shenzhen Center for Disease Control and Prevention, Shenzhen 518055, China; 5 Southwest United Graduate School, Kunming 650092, China

**Keywords:** flavivirus, noncoding RNA, subgenomic flaviviral RNAs, viral replication, vector-borne transmission, pathogenicity

## Abstract

Noncoding RNAs (ncRNAs) constitute a class of RNA molecules that lack protein-coding capacity. ncRNAs frequently modulate gene expression through specific interactions with target proteins or messenger RNAs, thereby playing integral roles in a wide array of cellular processes. The Flavivirus genus comprises several significant members, such as dengue virus (DENV), Zika virus (ZIKV), and yellow fever virus (YFV), which have caused global outbreaks, resulting in high morbidity and mortality in human populations. The life cycle of arthropod-borne flaviviruses encompasses their transmission between hematophagous insect vectors and mammalian hosts. During this process, a complex three-way interplay occurs among the pathogen, vector, and host, with ncRNAs exerting a critical regulatory influence. ncRNAs not only constitute a crucial regulatory mechanism that has emerged from the coevolution of viruses and their hosts but also hold potential as antiviral targets for controlling flavivirus epidemics. This review introduces the biogenesis of flavivirus-derived ncRNAs and summarizes the regulatory roles of ncRNAs in viral replication, vector-mediated viral transmission, antiviral innate immunity, and viral pathogenicity. A profound comprehension of the interplay between ncRNAs and flaviviruses will help formulate efficacious prophylactic and therapeutic strategies against flavivirus-related diseases.

## 1. Introduction

Within biological entities, a plethora of RNA molecules devoid of protein-coding capability, termed noncoding RNAs (ncRNAs), exist. These RNAs are classified into two principal categories based on transcript length: long noncoding RNAs (lncRNAs), which exceed 200 nucleotides (nt), and short noncoding RNAs (sncRNAs), which are under 200 nt. The latter encompasses microRNAs (miRNAs), small interfering RNAs (siRNAs), PIWI-interacting RNAs (piRNAs), small nuclear RNAs (snRNAs), vault RNAs (vtRNAs), and transfer RNA-derived small RNAs (tsRNAs), among others [1]. Advancements in technologies such as microarrays and high-throughput sequencing have led to the discovery of an increasing array of ncRNAs. Studies demonstrate that a mere fraction, less than 2%, of genes transcribe into messenger RNAs (mRNAs) with the capacity for protein translation, with the overwhelming majority being ncRNAs [2]. Initially deemed “junk” RNA, ncRNAs have been increasingly corroborated to exert significant regulatory functions within cellular responses. ncRNAs engage in interactions with DNA, RNA, and proteins, modulating gene expression at transcriptional and posttranscriptional stages by influencing chromatin remodeling, mRNA stability, and protein synthesis [3,4,5]. Furthermore, ncRNAs can bind directly to target proteins, influencing protein function and localization and thus playing pivotal roles in critical biological processes, including cell proliferation, differentiation, apoptosis, and immune responses [6,7,8]. The relationship between ncRNAs and viral replication, as well as pathogenesis, is important. Various pathogens, such as members of the *Adenoviridae*, *Herpesviridae*, and *Flaviviridae*, are capable of producing their ncRNAs [4]. These pathogen-derived ncRNAs operate in regulatory capacities akin to host ncRNAs, influencing protein functionality and stability, and thereby impacting viral propagation and immune evasion within the host organism.

The Flavivirus genus belongs to the family *Flaviviridae* and comprises a group of single-stranded, positive-sense RNA viruses. Arthropod-borne flaviviruses were initially discovered within arthropods and are naturally transmitted through the bites of hematophagous insects such as mosquitoes, ticks, and sandflies [9,10]. Over the past few decades, factors such as global warming, increased human mobility, and viral evolution have led to the expanding reach of flavivirus infections [11,12,13]. Several significant pathogens, including dengue virus (DENV), Zika virus (ZIKV), Japanese encephalitis virus (JEV), yellow fever virus (YFV), and tick-borne encephalitis virus (TBEV), have consecutively emerged and caused outbreaks of both emerging and re-emerging infectious diseases in various regions, posing a substantial threat to global public health. The flavivirus genome ranges from 9.2 to 11.0 kb in length, consisting of a 5′ noncoding region (5′ UTR), a 3′ noncoding region (3′ UTR), and an intervening single open reading frame (ORF) [14]. Beyond serving as a carrier of genetic information, viral RNA also plays a crucial role as a necessary regulator of the viral life cycle, orchestrating the complex interplay between the virus and its host and vector. This regulatory role is pivotal in modulating viral replication and the overall virulence of the virus. The flaviviral 5′ UTR is approximately 100 nt in size and contains a unique m7GpppAmpN1 cap structure, compared to other members of *Flaviviridae*, which protects viral RNA from degradation by 5′-3′ exonucleases [15,16]. The 5′ UTR also includes several functional structures, such as two conserved stem loops (SLA and SLB) and the terminal hairpin structure (cHP), mediating interactions between the viral genome and proteins/nucleic acids, and is closely associated with viral genome cyclization, RNA synthesis, and viral protein translation [17,18,19]. The flaviviral 3′ UTR varies from 400 to 700 nt, forming multiple complex secondary structures. It contains three structural domains: Domain I is the variable region, including two stem-loop structures (SL-1 and SL-2) and some repeat sequences; Domain II is more conserved, containing two dumbbell structures (DB1 and DB2) as well as multiple conserved sequences (CS) and conserved repeat sequences (RCS); and Domain III is highly conserved, comprising the conserved sequence 3′CS, a small hairpin structure (sHP), and the terminal stem loop (3′ SL) [20,21,22,23,24]. The 3′ UTR is vital for processes such as viral RNA synthesis, protein translation, virion assembly, and pathogenicity [25,26,27,28,29,30]. Furthermore, the 3′ UTR is also the primary source for the production of flavivirus-derived ncRNAs [31].

Arthropod-borne flaviviruses are subject to the regulatory effects of ncRNAs from hosts, vectors, and viruses, which orchestrate the intricate “pathogen–vector–host” interactions that govern viral proliferation, dissemination, and virulence (Table 1). This review highlights the role of ncRNAs in flaviviral replication, vector-mediated viral transmission, immune regulation, and disease manifestation. Furthermore, this review will discuss the potential of ncRNAs as a basis for developing novel prophylactic and therapeutic strategies against flavivirus infections.

## 2. Noncoding RNA Derived from Flaviviruses

Most viruses capable of producing ncRNAs are DNA viruses, making flaviviruses, as positive-strand RNA viruses, particularly unique in their ability to form two classes of ncRNAs: subgenomic flaviviral RNAs (sfRNAs) and viral small RNAs (vsRNAs) [93]. Although the viral genomic UTRs act as *cis*-acting ncRNAs, we will focus on the functions of the aforementioned two types of virus-derived *trans*-acting ncRNAs in this review. sfRNA represents the most extensively characterized ncRNA within flaviviruses, typically ranging from 300 to 500 nt in size, possessing a highly structured conformation, and exhibiting notable resistance to nucleases [94,95]. sfRNA production occurs concurrently with RNA replication, arising from the incomplete degradation of the viral 3′ UTR by the cellular 5′-3′ exoribonuclease 1 (XRN1) [96]. XRN1, a fundamental component of the cellular RNA decay pathway, degrades monophosphorylated mRNA in a 5′-3′ direction [97,98]. Flaviviral RNA has evolved mechanisms to evade XRN1 degradation, with SL-1 and SL-2 structures in the 3′ UTR Domain I (also known as xrRNA1 and xrRNA2), inhibiting XRN1-mediated cleavage, resulting in sfRNAs of various lengths [99,100]. The structures of xrRNA1 and xrRNA2 are conserved across different flavivirus species [93,101,102]. sfRNAs are highly abundant in the cytoplasm, with levels several orders of magnitude higher than those of genomic RNAs during DENV infection [32]. sfRNA is dispensable for viral replication; mutants of Kunjin virus (KUNV), YFV, and DENV lacking the ability to produce sfRNAs still retain viral replication capability [31,39,103]. However, sfRNAs modulate a variety of cellular processes to ensure optimal viral adaptability and are closely related to immune antagonism and disease occurrence.

During flaviviral replication, an assortment of viral small RNAs (vsRNAs), measuring 10–30 nucleotides in length, is produced. This category of vsRNAs includes viral siRNAs and virus-encoded miRNAs. The viral replication process involves the liberation of viral genomic RNA into the cytoplasm, whereupon viral RNA-dependent RNA polymerase (RdRp) employs circularized positive-strand RNA as a template to synthesize complementary negative-strand RNA. This synthesis leads to the formation of double-stranded RNA (dsRNA) intermediates. The inherent hairpin structures within the UTR enable these dsRNAs to be recognized and processed by ribonucleases such as Dicers, resulting in their cleavage into vsRNA fragments [104]. In addition to this canonical pathway, sfRNAs also act as templates for the generation of vsRNAs through atypical routes [49,50]. RNA interference (RNAi) represents a critical antiviral pathway, wherein vsRNAs associate with Argonaute-2 (Ago2) proteins to form the RNA-induced silencing complex (RISC), targeting viral RNA for specific degradation and thus impeding viral replication [105]. Nevertheless, viruses frequently coopt host cellular machineries for their propagation, manipulating vsRNAs to modulate gene expression within both the viral genome and host cellular context. Several studies have elucidated the contributory roles of vsRNAs in the modulation of viral replication and immune responses, suggesting a repertoire of vsRNA functionalities extending beyond the conventional RNAi pathway [47,48,50]. The functional scope and mechanistic insights of vsRNAs remain, as of now, a subject of considerable debate and ongoing investigation.

## 3. Noncoding RNAs Regulate the Replication of Flavivirus

Upon entering cells, flaviviruses undergo nucleocapsid uncoating and release their genomic RNAs, subsequently proceeding through stages of protein translation, RNA replication, and virion assembly, culminating in the amplification of progeny virions. Accumulating evidence suggests that ncRNAs interact with viral and cellular proteins/RNAs during these processes, thereby directly or indirectly influencing virus proliferation. During West Nile virus (WNV) infection, sfRNAs are known to augment intracellular viral replication. These sfRNAs engage in a competitive interaction with Dicer’s dsRNA substrates, suggesting their role in facilitating viral replication by suppressing the RNAi antiviral pathway [39]. During ZIKV infections, sfRNAs bind various intracellular RNA-binding proteins, including several antiviral factors linked to RNA decay (such as DEAD-box helicase 6 [DDX6] and enhancer of mRNA decapping 3 [EDC3]) and RNA splicing (such as phosphorylated adaptor for RNA export [PHAX] and splicing factor 3b subunit 1 [SF3B1]), ultimately attenuating the cellular antiviral response [33]. sfRNAs are also implicated in inhibiting XRN1, leading to an accumulation of uncapped transcripts and a concomitant destabilization of mRNA homeostasis, which diminishes the resistance of cells to DENV and KUNV infections [34]. In another study, a miRNA derived from the KUNV 3′ SL region, termed KUN-miR-1, was shown to associate with GATA binding protein 4 (GATA4) mRNA, enhancing its expression and viral replication [49]. In addition to the roles mentioned above, sfRNAs and vsRNAs contribute to the autoregulatory mechanisms of viral replication. During JEV infection, the presence of sfRNAs correlates with the plateau phase of negative-strand RNA synthesis. Empirical evidence indicates that the accumulation of sfRNAs restricts the synthesis of negative-strand RNA and thereby moderates viral RNA synthesis and protein translation [45]. Moreover, a distinct miRNA produced post-DENV infection, designated DENV-vsRNA-5, specifically targets viral nonstructural protein 1 (NS1), constraining viral replication. The selective inhibition of DENV-vsRNA-5 markedly escalates viral replication levels [50].

Host- and vector-derived ncRNAs also exert regulatory control over viral infections [106]. Certain miRNAs modulate viral replication by directly targeting viral genomic RNAs. For instance, miR-548g-3p is upregulated during DENV infection, targeting the DENV 5′ UTR and suppressing viral amplification [51]. In contrast, miR-133A, miR-484, and miR-744 are downregulated upon DENV infection; these miRNAs bind to the DENV 3′ UTR, inhibiting viral replication [54,55]. Additionally, the *Aedes aegypti* miRNA miR-252 targets gene coding for the DENV envelope protein (E), reducing its expression and consequently impeding viral replication [56]. Alternatively, a distinct class of miRNAs influences viral infection indirectly by modulating host factors. miR-142-5p impairs the expression levels of the integrin subunit alpha V (ITGAV), which assists the virus in internalizing into cells, thereby suppressing ZIKV replication [67]. miR-103a-3p stimulates the p38 mitogen-activated protein kinase (MAPK) signaling pathway by targeting the gene OTU deubiquitinase 4 (OTUD4), consequently leading to enhanced replication of ZIKV [52]. miR-383-5p suppresses the expression of the phospholipase A2 group IVA (PLA2G4A), a host factor crucial for the production of infectious DENV in hepatic cells [53,107]. miR-532-5p targets the SEC14 and spectrin domain containing 1 (SESTD1) and TGF-β activated kinase 1 and MAP3K7 binding protein 3 (TAB3) genes, inhibiting their expression and thus constraining KUNV replication [57]. Furthermore, Hs_154 downregulates CCCTC-binding factor (CTCF) and EGFR-coamplified and overexpressed protein (ECOP) gene expression, significantly attenuating viral replication by enhancing WNV-induced apoptosis [58]. miRNAs may exhibit differential regulatory effects across diverse viruses. Specifically, miR-21 enhances the replication of DENV while exhibiting antiviral effects against ZIKV [59,60]. Further investigation is required to elucidate the mechanisms by which miR-21 regulates flaviviral replication. Recent research increasingly highlights the crucial role of cellular lncRNAs in host–pathogen interactions. In murine neuronal cells infected with JEV, lncRNA-MALAT1 is markedly upregulated through the PKR-like ER kinase (PERK) endoplasmic reticulum stress signaling pathway [87]. Additionally, swine lncRNA-SUSAJ1 has been documented to inhibit JEV replication, suggesting its potential role as an integral component of the cellular antiviral defense mechanism [88].

## 4. The Roles of Noncoding RNAs in Vector-Mediated Flavivirus Infections

**Figure 1:**
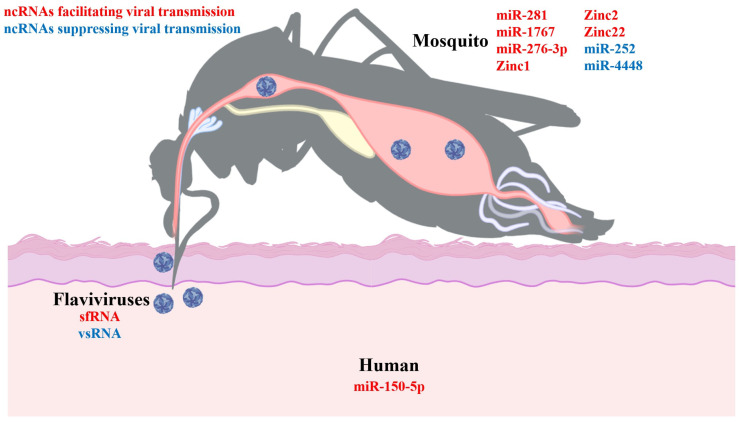
Effects of noncoding RNAs on flavivirus transmission by mosquitoes. Mosquitoes may acquire viruses by biting patients infected with flavivirus. After traversing the midgut, the viruses enter the hemolymph and eventually reach the salivary glands, where the virus could be released into a healthy individual during a bite. Throughout this process, noncoding RNAs (ncRNAs) derived from the virus, humans, and mosquitoes play a crucial regulatory role. These ncRNAs either act directly on the viral genome, influencing viral replication, or modulate the RNA interference (RNAi) antiviral pathway in the mosquito.

Mosquitoes are the principal vectors for flavivirus transmission, playing an integral role in the viral life cycle. Viral acquisition by mosquitoes occurs through blood feeding from infected hosts. The virus must overcome the midgut barrier to establish infection within the gut epithelial cells and subsequently spread to the hemolymph. Flaviviruses traverse the hemolymph to infect various mosquito tissues, including fat bodies, ovaries, salivary glands, and the nervous system, facilitating transmission to new hosts during subsequent feeding events through salivary secretions [108,109,110,111]. Throughout evolutionary history, flaviviruses have utilized ncRNAs as a strategy to antagonize and circumvent the antiviral defenses of mosquitoes (Figure 1). The production of substantial amounts of sfRNAs within the mosquito facilitates the efficient proliferation and transmission of flaviviruses. It has been observed that the absence of sfRNA impedes WNV infectivity in mosquitoes when delivered via blood feeding. In contrast, the direct intrathoracic inoculation of viruses does not demonstrate this effect, indicating that the presence of sfRNA markedly improves the efficiency of the viral passage across the midgut barrier [40]. Numerous studies support the facilitative role of sfRNA in the propagation of WNV, DENV, and other flaviviruses within mosquito cells by modulating the RNAi machinery—an essential antiviral defense in insects [39,112]. sfRNA is known to function as a molecular decoy for Dicer and Ago2, thus attenuating the RNAi pathway [112]. Moreover, the regulatory impact of sfRNA on mosquito gene expression, particularly in genes governing apoptosis, has been demonstrated to foster an environment conducive to ZIKV replication by suppressing cell death mechanisms in infected tissues [42]. In addition to enhancing viral fitness within mosquitoes, sfRNA aids in the transmission of the virus from the vector to the host. A recent study indicates that salivary extracellular vesicles of DENV-infected mosquitoes contain sfRNAs. These sfRNAs assist the virus in establishing infection in cells at the mosquito bite site by inhibiting interferon (IFN) induction and signaling [35]. A comparative study suggests that flaviviruses generate a greater quantity of vsRNAs in mosquito cells than in mammalian cells [47]. In insects, various sizes of vsRNAs are produced through different antiviral pathways. In *Aedes aegypti* mosquitoes and *Aedes aegypti*-derived Aag2 cells, DENV infection induces the formation of approximately 21 nt vsRNAs, which are products of the RNAi pathway [48]. In C6/36 cells, an RNAi-deficient cell line derived from *Aedes albopictus*, DENV infection results in the production of a distinct class of approximately 27 nt vsRNAs, subsequently confirmed to be generated through the PIWI pathway [113]. These vsRNAs play crucial roles in inhibiting viral infection in the vector, and further research is needed to determine whether they have additional functions.

Vector-derived ncRNAs are implicated in modulating the interaction between arthropod vectors and viruses. miR-252 of *Aedes albopictus* is markedly upregulated after DENV infection and has been shown to reduce the viral RNA load and expression of the viral E protein [56]. Conversely, miR-281, a miRNA with midgut-specific expression in *Aedes aegypti*, is upregulated following DENV infection and interacts with the 5′ UTR of the viral genome, consequently enhancing viral replication and dissemination [62]. Another study identified 46 altered miRNA expressions in the *Aedes aegypti* midgut post-DENV infection, with miR-1767 and miR-276-3p enhancing DENV replication in C6/36 cells, while miR-4448 has the opposite effect [63]. A recent study has disclosed that ZIKV infection significantly regulates the transcript levels of six lncRNAs in *Aedes aegypti*. Notably, the silencing of three lncRNAs (designated Zinc1, Zinc2, and Zinc22), results in a marked reduction in mosquito susceptibility to ZIKV infection [89].

During hematophagy, arthropods acquire various factors, including components from host blood and metabolites from commensal microbes, which might influence viral transmission [114,115,116,117,118]. A recent finding revealed that mosquitoes acquire a host-derived miRNA, known as miR-150-5p, which has been shown to downregulate the expression of the thrombospondin-related anonymous protein AaCT-1 mRNA, thereby disrupting the RNAi antiviral framework and promoting DENV infection and propagation in the mosquito [36].

## 5. Involvement of Noncoding RNAs in Innate Immunity against Flaviviruses

The innate immune system constitutes a formidable barrier against virus infection. During a typical flavivirus infection, exogenous viral nucleic acids are recognized by various pattern recognition receptors (PRRs), triggering an IFN response and activating a cascade of interferon-stimulated genes (ISGs). This leads to the production of proinflammatory cytokines and the induction of an antiviral state within the cell [119]. Studies have demonstrated that sfRNA, as a key regulatory factor, can subvert host immune defenses, enabling more efficient viral replication and severe infection. For instance, WNV sfRNAs assist in the viral evasion of type I IFN-mediated antiviral responses [41]. DENV sfRNAs interact with the tripartite motif containing 25 (TRIM25) protein, preventing its deubiquitination—a process crucial for activating the retinoic acid inducible gene-I (RIG-I)-mediated IFN response—thereby inhibiting the translation of ISGs [32]. Furthermore, DENV sfRNAs bind to G3BP stress granule assembly factor 1 (G3BP1), G3BP stress granule assembly factor 2 (G3BP2), and cell cycle associated protein 1 (CAPRIN1), disrupting the translation of antiviral ISGs [37]. Research on JEV has shown that sfRNAs impede the phosphorylation and nuclear localization of interferon regulatory factor-3 (IRF-3), thereby downregulating downstream IFN-β promoter activity and IFN-β mRNA levels. This results in a reduction in IFN-β-induced apoptosis, supporting the role of sfRNA in counteracting the host antiviral response and aiding in the establishment of persistent viral infection [46]. A study indicated that WNV generates a higher quantity of vsRNAs in mice lacking type I interferon receptors (IFNAR), with vsRNA levels inversely correlated with the IFN response [120]. Conversely, DENV produces more vsRNAs in Huh7 cells (human hepatocarcinoma cell line), which possess a complete IFN pathway, than in IFN-deficient Vero cells (kidney epithelial cells from African green monkey) [47]. These findings imply a potential association between vsRNAs and IFN signaling.

Host organisms harbor numerous miRNA-targeting genes contributing to innate immunoregulation. Upon viral infection, numerous miRNAs are regulated, thereby bolstering the antiviral defense mechanisms to counteract the viral invasion. It has been observed that miR-424 is upregulated during DENV infection, which suppresses the expression of the siah E3 ubiquitin protein ligase 1 (SIAH1), preventing the ubiquitination and degradation of the Toll-like receptor (TLR) signaling adaptor protein MyD88. This facilitates the stimulation of Toll-like receptor 7 (TLR7)-associated antiviral immune signaling [64]. Furthermore, DENV infection induces the upregulation of Let-7c and miR-30e*, wherein Let-7c plays a crucial role in modulating the oxidative stress response and miR-30e* triggers IFN-β signaling via the nuclear factor kappa-B (NF-κB)-dependent pathway, ultimately leading to the suppression of viral replication [61,76]. Additionally, ZIKV infection significantly reduces the cellular levels of miR-142-5p, which has been demonstrated to inhibit the expression of the IL-6 signal transducer (IL6ST); IL6ST is associated with T cell response and the activation of the Janus kinase/signal transducer and activator of transcription (JAK/STAT) pathway, playing a role in antiviral defense [67]. During infection, flaviviruses strategize to suppress host miRNAs associated with antiviral responses, thereby facilitating immune evasion. DENV infection downregulates miR-155, which targets BTB domain and CNC homolog 1 (BACH1), leading to the inhibition of viral protease activity, and thereby aiding in the induction of IFN responses [68]. Duck Tembusu virus (DTMUV) infection downregulates miR-148a-5p and upregulates miR-221-3p, which are known to target the suppressor of cytokine signaling 1 (SOCS1) and the suppressor of cytokine signaling 5 (SOCS5), respectively, resulting in significantly reduced production of type I IFNs [65,66,121]. JEV infection leads to the downregulation of miR-432, which plays a role in triggering the antiviral inflammatory response by modulating SOCS5 expression and signal transducer and activator of transcription 1 (STAT1) phosphorylation [75]. Aside from miRNAs with antiviral activity, certain host miRNAs may antagonize immune responses, thereby assisting viral infection. miR-146a is upregulated in cells infected with DENV, ZIKV, and JEV, which can downregulate the expression of the TNFR-associated factor 6 (TRAF6) and STAT1 genes, thereby suppressing the proinflammatory and antiviral pathways regulated by these two genes [77,78,79]. Cellular lncRNA is involved in modulating immune responses, thereby influencing viral replication and pathogenicity. Specifically, in neuronal cells infected with JEV, a notable increase in the expression of lncRNA-JINR1 is observed. LncRNA-JINR1 interacts with RNA binding motif protein 10 (RBM10) and NF-κB, consequently promoting viral replication and virus-induced apoptosis [90].

## 6. Noncoding RNAs and Their Implications in Flaviviral Disease Pathogenesis

Over 50% of known flaviviruses have been reported to be associated with human diseases, often manifesting symptoms that include central nervous system disorders (such as meningitis and encephalitis), fever, hemorrhagic fever, arthralgia, etc. ncRNAs play a significant role in the pathogenesis of virus-induced diseases [122,123,124]. The function of sfRNA in regulating virus-induced cytopathicity and pathogenicity has been elucidated in DENV, YFV, ZIKV, and WNV. For instance, DENV sfRNAs have been reported to inhibit protein kinase B (Akt) phosphorylation via a BCL-2-dependent mechanism, promoting apoptosis by impeding the phosphoinositide 3-kinase and protein kinase B (PI3K-Akt) survival pathway [38]. Conversely, JEV sfRNAs inhibit IFN-β-stimulated apoptosis, aiding in viral persistence [46]. sfRNAs are also implicated in neurogenesis. A prominent pathology associated with ZIKV is microcephaly in fetuses [125]. Utilizing a murine pregnancy model, researchers have determined that ZIKV sfRNAs are crucial for the virus to penetrate the maternal placental barrier, facilitating ZIKV infection of the fetal brain. This study has demonstrated that ZIKV sfRNAs bind and stabilize viral nonstructural protein 5 (NS5) in human placental cells, leading to reduced phosphorylation and nuclear translocation of STAT1, ultimately inhibiting various antiviral pathways and promoting apoptosis [43]. Additionally, sfRNAs induce apoptosis in neural progenitor cells within human cerebral organoids, leading to their disintegration. Recent research employing a human 3D cerebral organoid model to explore the role of ZIKV sfRNAs in neural cell pathogenesis has found that sfRNAs significantly downregulate the expression of genes related to neural differentiation, potentially through interference with the Wnt signaling and proapoptotic pathways [44].

Meanwhile, cellular miRNAs play a vital role in flaviviral pathogenesis. WNV infection in murine brain tissues leads to a significant reduction in the levels of miR-196a, miR-202-3p, miR-449c, and miR-125a-3p, which target cytokines, chemokines, and apoptotic genes involved in WNV-induced neuropathogenesis [69]. Neuronal infection by JEV/WNV leads to the overexpression of miR-451a, which in turn promotes virus-induced neuronal cell death by regulating the 14-3-3ζ-JNK axis [80]. miR-155, known to regulate various stages of the innate immune response during virus-induced inflammation, is upregulated during JEV, WNV, and ZIKV infections. Several studies have revealed that miR-155 plays multiple roles in viral pathogenesis and cell survival by modulating pathways related to neuroinflammation [69,70,71,72,73,74]. Furthermore, a genome-wide integrative analysis has indicated that ZIKV infection of human neural stem cells (hNSCs) results in the differential expression of a suite of miRNAs associated with microcephaly, including the upregulation of miR-124-3p, which represses transferrin receptor (TFRC) mRNA, impeding NSC proliferation [81]. ZIKV E protein upregulates miR-204-3p and miR-1273g-3p, which target neurogenic locus notch homolog protein 2 (NOTCH2) and paired box 3 (PAX3), thereby modulating the differentiation and apoptosis of NSCs [82]. Additional studies have identified several miRNAs, such as miR34c, miR-145, miR-9, and miR-148a, which play roles in the growth of NSCs and the development of congenital Zika syndrome, that were upregulated following ZIKV infection [83,84,85]. Certain ncRNAs have the potential to serve as biomarkers indicative of disease progression. miR-96-5p and miR-146a-5p have been observed to undergo dysregulation in patients suffering from severe dengue disease. These two exosomal miRNAs are implicated in targeting a range of genes that are closely associated with the immune and inflammatory response [86]. RNA-seq analysis has revealed that in peripheral blood mononuclear cells of patients infected with dengue, lncRNA NEAT1 is negatively correlated with the severity of the disease [91]. Correspondingly, another study has demonstrated that the knockdown of NEAT1 leads to the increased expression of interferon alpha-inducible protein 27 (IFI27), thereby promoting antiviral defense through the RIG-I pathway [92].

## 7. Conclusions and Perspectives

Arthropod-borne flaviviruses are involved in transmission cycles characterized by intricate three-way interplay among the pathogen, vector, and host. Future investigations will need to utilize the advancements in next-generation sequencing and bioinformatics to elucidate the role of ncRNAs in these complex interactions. A comprehensive understanding of how flaviviruses employ ncRNAs to modulate gene expression and enhance viral adaptability, as well as how hosts leverage ncRNAs to resist infection, will significantly deepen our insight into flavivirus transmission dynamics in nature. Despite the widespread prevalence of flaviviruses, effective antiviral therapies are lacking, and vaccines are available for only a few flaviviruses, such as YFV and JEV. Insights into the ncRNA-mediated regulation of flavivirus propagation and pathogenicity will facilitate the development of novel preventive strategies and control measures to curb flavivirus outbreaks. Due to the base-pairing mechanisms of ncRNAs, they are amenable to being designed as therapeutic targets against precise viral sequences, offering advantages over traditional targets. Their pivotal involvement in viral replication, immune regulation, and disease pathogenesis positions ncRNAs as promising candidates for novel prophylactic and therapeutic modalities. Therapeutic approaches include employing antiviral ncRNAs to inhibit viral replication or designing inhibitors to obstruct the production of ncRNAs that facilitate viral transmission, thereby averting viral immune evasion. The clinical efficacy of several antisense oligonucleotide (ASO) and ASO anti-microRNA (antimiR) therapeutics targeting various viruses, such as Cytomegalovirus (CMV) and Hepatitis C Virus (HCV), has been validated in clinical trials [126]. Notably, RNA viruses have a high propensity to accumulate adaptive mutations, leading to the emergence of drug-resistant strains. Therefore, it is imperative to explore synergistic strategies that combine ncRNA therapeutics with other pharmaceutical interventions. Moreover, the development of drugs targeting multiple ncRNAs, as well as universal antiviral ncRNA therapeutics that modulate host innate immune responses and inflammatory pathways, represents a promising avenue for future research. In the field of vaccine development and disease diagnostics, ncRNAs also have significant roles. The insertion of immunoregulatory miRNAs into the noncoding regions of the flaviviral genome has been recognized as an effective technique for generating live attenuated vaccines. Moreover, ncRNAs present in the serum are being explored as potential diagnostic biomarkers for indicating disease severity. In conclusion, ncRNAs are instrumental in adjusting the equilibrium between flavivirus-induced pathogenicity and host antiviral defense. Broad and detailed research is essential to better define the functionality of ncRNAs within the context of flavivirus biology and to accelerate the progression of therapeutic interventions.

## Figures and Tables

**Table 1:** Noncoding RNAs involved in flaviviruses infection.

ncRNAs	Virus(es)	Targets	Functions	References
sfRNA	DENV	XRN1, DDX6, EDC3, PHAX, SF3B1, Dicer, Ago2, TRIM25, G3BP1, G3BP2, CAPRIN1, Akt	dysregulate host mRNA stability; inhibit RNAi machinery; antagonize type I IFN-mediated antiviral responses; disrupt the translation of antiviral ISGs; promote virus-induced apoptosis	[32,33,34,35,36,37,38]
sfRNA	WNV, KUNV	XRN1, Dicer	dysregulate host mRNA stability; inhibit RNAi machinery; assist the virus in overcoming the mosquito midgut barrier; antagonize type I IFN-mediated antiviral responses	[34,39,40,41]
sfRNA	ZIKV	DDX6, EDC3, PHAX, SF3B1, CASP7, Viral NS5	dysregulate host mRNA stability; facilitate virus transmission by inhibiting cell death in mosquito tissues; inhibit type I and type III IFN signaling; promote apoptosis of mammalian cells; impair brain development by interfering with Wnt-signaling and proapoptotic pathways	[33,42,43,44]
sfRNA	JEV	Viral antigenome, IRF-3	inhibit viral antigenome synthesis and protein translation; reduce IFN-β-stimulated apoptosis	[45,46]
vsRNA	DENV, WNV	Viral genome	suppress viral replication; possible association with IFN signaling pathway	[47,48]
KUN-miR-1	KUNV	GATA4	facilitate viral replication	[49]
DENV-vsRNA-5	DENV	Viral NS1	suppress viral replication	[50]
miR-548g-3p	DENV	Viral 5′UTR	interfere with viral protein translation and suppress viral replication	[51]
miR-103a-3p	ZIKV	OTUD4	facilitate viral replication	[52]
miR-383-5p	DENV	PLA2G4A	suppress viral replication	[53]
miR-133a	DENV	Viral 3′UTR	suppress viral replication	[54]
miR-484	DENV	Viral 3′UTR	suppress viral replication	[55]
miR-744	DENV	Viral 3′UTR	suppress viral replication	[55]
miR-252	DENV	Viral E	suppress viral replication	[56]
miR-532-5p	KUNV	SESTD1, TAB3	suppress viral replication	[57]
Hs_154	WNV	CTCF, ECOP	enhance viral-induced apoptosis and inhibit viral replication	[58]
miR-21	DENV, ZIKV		modulate viral replication	[59,60]
Let-7c	DENV	BACH1	induce the anti-oxidative and anti-inflammatory response	[61]
miR-281	DENV	Viral 5′UTR	enhance virus infection in midgut of mosquitoes	[62]
miR-1767	DENV		facilitate viral replication	[63]
miR-276-3p	DENV		facilitate viral replication	[63]
miR-4448	DENV		suppress viral replication	[63]
miR-150-5p	DENV	AaCT-1	disrupt RNAi system and promote virus transmission in the mosquito	[36]
miR-424	DENV	SIAH1	facilitate TLR signaling activated cellular defenses	[64]
miR-148a-5p	DTMUV	SOCS1	promote IFN-α/β production	[65]
miR-221-3p	DTMUV	SOCS5	inhibit IFN-β production	[66]
miR-142-5p	ZIKV	IL6ST, ITGAV	modulate (inhibit) JAK/STAT signaling pathway; suppress virus binding to cells;	[67]
miR-155	DENV, JEV, WNV, ZIKV	BACH1, PELI1, SHIP1	promote IFN-α production; modulate virus-induced inflammatory response	[68,69,70,71,72,73,74]
miR-432	JEV	SOCS5	promote JAK/STAT signaling pathway	[75]
miR-30e *	DENV	IκBα 3′UTR	promote IFN-β production	[76]
miR-146a	DENV, ZIKV, JEV	TRAF6, STAT1	suppress proinflammatory and innate antiviral immunity	[77,78,79]
miR-196a	WNV	CCR2, NFKBIA, SMAD6	suppress viral-induced neuroinflammation	[69]
miR-202-3p	WNV	TNFRSF1B, CCR7, BCL2L1, S100A8, THBS1, CCL7, IL10	suppress viral-induced neuroinflammation	[69]
miR-449c	WNV	CXCL10, CXCL11, NFKBIA, SERPINE1, IL2RB, CCR1, MYC, SNAI1, BCL6	suppress viral-induced neuroinflammation	[69]
miR-125a-3p	WNV	PTGS2, IL1R1, IL10, CCL4	suppress viral-induced neuroinflammation	[69]
miR-451a	JEV, WNV	14-3-3ζ	induce neuronal apoptosis	[80]
miR-124-3p	ZIKV	TFRC	dysregulate NSC maintenance	[81]
miR-204-3p	ZIKV	NOTCH2	impair NSC proliferation and differentiation	[82]
miR-1273g-3p	ZIKV	PAX3	impair NSC proliferation and differentiation	[82]
miR34c	ZIKV	BCL2, NOTCH, NUMB	reduce NSC and GSC cell growth	[83]
miR-145	ZIKV	CDH2, ACTG1, ACTB, CDK6	impair cell migration; involved in CNS formation	[84]
miR-9	ZIKV	GDNF	induce neuronal apoptosis	[85]
miR-148a	ZIKV	MDFIC, SNX27, SKP1,	impair cell migration; involved in CNS formation	[84]
miR-96-5p	DENV		regulate immune and inflammatory responses	[86]
miR-146a-5p	DENV		regulate immune and inflammatory responses	[86]
MALAT1	JEV		regulate host cell death	[87]
SUSAJ1	JEV		suppress viral replication	[88]
Zinc1, Zinc2, Zinc22	ZIKV		facilitate viral infection in mosquitoes	[89]
JINR1	JEV, DENV	RBM10	facilitate viral replication, induce neuronal cell death	[90]
NEAT1	DENV	IFI27	suppress antiviral response via the RIG-I pathway	[91,92]

The asterisk (*) indicates passenger strand. Abbreviations: DENV, dengue virus; WNV, West Nile virus; KUNV, Kunjin virus; ZIKV, Zika virus; JEV, Japanese encephalitis virus; DTMUV, duck Tembusu virus; E, envelope protein; NS, nonstructural protein; UTR, untranslated region; XRN1, 5′-3′ exoribonuclease 1; DDX6, DEAD-box helicase 6; EDC3, enhancer of mRNA decapping 3; PHAX, phosphorylated adaptor for RNA export; SF3B1, splicing factor 3b subunit 1; Ago2, Argonaute-2; TRIM25, tripartite motif containing 25; G3BP1, G3BP stress granule assembly factor 1; G3BP2, G3BP stress granule assembly factor 1; CAPRIN1, cell cycle associated protein 1; Akt, protein kinase B; CASP7, caspase 7; IRF-3, interferon regulatory factor 3; GATA4, GATA binding protein 4; OTUD4, OTU deubiquitinase 4; PLA2G4A, phospholipase A2 group IVA; SESTD1, SEC14 and spectrin domain containing 1; TAB3, TGF-β activated kinase 1 and MAP3K7 binding protein 3; CTCF, CCCTC-binding factor; ECOP, EGFR-coamplified and overexpressed protein; BACH1, BTB domain and CNC homolog 1; SIAH1, siah E3 ubiquitin protein ligase 1; SOCS, the suppressor of cytokine signaling; IL, interleukin; IL6ST, IL-6 signal transducer; ITGAV, integrin subunit alpha V; PELI1, pellino E3 ubiquitin protein ligase 1; SHIP1, inositol polyphosphate-5-phosphatase D; TRAF6, TNFR-associated factor 6; CCR, C-C motif chemokine receptor; CCL, C-C motif chemokine ligand; CXCL, C-X-C motif chemokine ligand; NFKBIA, NF-Kappa-B inhibitor alpha; SMAD6, SMAD family member 6; TNFRSF1B, TNF receptor superfamily member 1B; BCL2, BCL2 apoptosis regulator; BCL2L1, BCL2 like 1; BCL6, BCL6 transcription repressor; S100A8, S100 calcium binding protein A8; THBS1, thrombospondin 1; SERPINE1, serpin family E member 1; IL2RB, interleukin 2 receptor subunit beta; MYC, MYC proto-oncogene, BHLH transcription factor; SNAI1, snail family transcriptional repressor 1; PTGS2, prostaglandin-endoperoxide synthase 2; IL1R1, interleukin 1 receptor type 1; TFRC, transferrin receptor; NOTCH2, notch homolog protein 2; PAX3, paired box 3; NUMB, NUMB endocytic adaptor protein; CDH2, cadherin 2; ACTG1, actin gamma 1; ACTB, actin beta; CDK6, cyclin dependent kinase 6; GDNF, glial cell derived neurotrophic factor; MDFIC, MyoD family inhibitor domain containing; SNX27, sorting nexin 27; SKP1, S-phase kinase associated protein 1; RBM10, RNA binding motif protein 10; IFI27, interferon alpha-inducible protein 27; RNAi, RNA interference; IFN, interferon; ISG, interferon-stimulated gene; STAT1, signal transducer and activator of transcription 1; TLR, Toll-like receptor; RIG-I, retinoic acid inducible gene-I; JAK/STAT, Janus kinase/signal transducer and activator of transcription; NSC, neural stem cell; GSC, glioma stem cells; CNS, central nervous system.
